# Supplementary material for: Sensitivity of Nutrition Indicators to Measure the Impact of a Multi-Sectoral Intervention: Cross-Sectional, Household, and Individual Level Analysis
Source: Int J Environ Res Public Health. 2020 Apr 30;17(9):3121. doi: 10.3390/ijerph17093121 (PMC7246654; doi:10.3390/ijerph17093121)
Supplement: Supplementary file 1 [file ijerph-17-03121-s001.pdf]

Supplemental Tables

Table S1. Sample size by type of analysis, nutrition outcome, and control vs treatment.

	Year	Treatment			Control			Total		
Household data	*2012*	700			718			1418		
	2014	608			638			1246		
	2015	627			632			1259		
	**Total**	**1,935**			**1988**			**3,923**		
Child data		**WAZ**	**HAZ**	**WHZ**	**WAZ**	**HAZ**	**WHZ**	**WAZ**	**HAZ**	**WHZ**
Non-matched	*2012*	628	617	613	665	650	648	1,293	1267	1,261
	2014	560	549	553	581	568	572	1,141	1117	1,125
	2015	517	514	512	571	661	564	1,088	1075	1,076
	**Total**	**1705**	**1680**	**1678**	**1817**	**1779**	**1784**	**3522**	**3469**	**3462**
Matched on the *household*	*2012*	404	405	399	448	441	442	852	846	841
	2014	351	347	348	371	368	366	722	715	714
	2015	341	340	339	362	360	360	703	700	699
	**Total**	**1096**	**1092**	**1086**	**1181**	**1169**	**1168**	**2277**	**2261**	**2254**
Matched on the *child*	*2012*	175	172	171	173	173	172	348	345	343
	2014	303	299	302	292	288	287	595	587	589
	2015	272	271	270	290	284	285	562	555	555
	**Total**	**750**	**742**	**743**	**755**	**745**	**744**	**1505**	**1487**	**1487**

Table S2. Number of households with one child and more than one child (%) with anthropometry data across the treatment group and time.

	Treatment			Control		
	1 child	> 1 child		1 child	> 1 child	
	n	N	%	N	n	%
2012	200	199	49%	247	195	44%
2014	164	184	53%	180	186	50%
2015	180	159	47%	183	177	49%
**Total**	**544**	**542**	**50%**	**610**	**558**	**48%**

Table S3. Descriptive statistics on nutrition outcome indicators across matched and non-matched children over time.

	2012		2014		2015	
**Indicator**	**Not-matched**	**matched**	**Not-matched**	**matched**	**Not-matched**	**matched**
wasting (%)	15.7 (12.5; 19.0)	14.1 (10.1; 18.1)	16.2 (12.2; 20.3)	12.8 (8.9; 16.6)	18.5 (13.8; 23.2)	15.6 (11.8; 19.4)
Severe wasting (%)	2.6 (1.5; 3.8)	3.5 (1.5; 5.5)	4.1 (1.8; 6.4)	3.2 (1.3; 5.1)	5.4 (0.6; 10.2)	3.8 (2.0; 5.5)
WHZ	-0.90 (-0.99; -0.81)	-0.90 (-1.05; -0.75)	-0.91 (-1.03; -0.78)	-0.86 (-0.97; -0.74)	-0.97 (-1.17; -0.78)	-0.96 (-1.08; -0.83)
stunting (%)	37.9 (33.0; 42.6)	39.2 (32.8-45.5)	42.8 (37.3-48.2)	44.9 (39.7-50.2)	39.5 (34.3-44.8)	41.6 (36.2-47.0)
HAZ	-1.42 (-1.59; -1.25)	-1.51 (-1.72; -1.30)	-1.72 (-1.91; -1.52)	-1.83 (-1.98; -1.67)	-1.53 (-1.65; -1.41)	-1.66 (-1.82; -1.49)
underweight (%)	29.9 (25.6; 34.1)	32.9 (26.3; 39.5)	36.7 (31.0; 42.5)	35.5 (30.5; 40.6)	30.7 (26.3; 35.1)	36.0 (30.6; 41.4)
WAZ	-1.42 (-1.55; -1.30)	-1.48 (-1.63; -1.33)	-1.66 (-1.79; -1.53)	-1.64 (-1.73; 1.54)	-1.54 (-1.67; -1.40)	-1.61 (-1.74; -1.48)

‡ mean with 95% confidence intervals in parentheses (controlling for population and design effect)
* p-value<0.05; ** p-value<0.01; *** p-value<0.001 (within time across group comparison)

Table S4. Descriptive statistics on nutrition outcome indicators by time and treatment.

	2012		2014		2015	
**Indicator**	**Treatment**	**control**	**treatment**	**Control**	**treatment**	**control**
wasting (%)	15.0 (10.9; 19.1)	15.6 (12.4; 18.7)	15.6 (11.6; 19.7)	13.5 (8.8; 18.1)	13.7 (9.5; 18.0)	19.5 (15.6; 23.5)
Severe wasting (%)	3.6 (2.0; 5.3)	2.1 (0.9; 3.3)	3.2 (1.3; 5.0)	4.0 (1.9; 6.1)	2.6 (0.2; 5.1)	6.0 (3.0; 9.0)
WHZ	-0.88 (-1.00; -0.76)	-0.91 (-1.02; -0.81)	-0.89 (-0.99; -0.79)	-0.87 (-1.02; -0.72)	**-0.80* (-0.98; -0.63)**	**-1.09 (-1.24; -0.94)**
stunting (%)	36.8 (29.2; 44.5)	39.4 (34.4-44.4)	45.9 (39.2-52.6)	42.2 (37.5-47.0)	37.6 (32.2-43.1)	42.9 (36.8-49.0)
HAZ	-1.40 (-1.62; -1.18)	-1.48 (-1.67; -1.29)	-1.88 (-2.11; -1.65)	-1.68 (-1.82; -1.54)	-1.57 (-1.70; -1.44)	-1.61 (-1.77; -1.46)
underweight (%)	29.8 (24.6; 35.0)	31.2 (26.6; 35.9)	35.6 (28.8; 42.5)	36.5 (32.5; 40.6)	**28.7* (23.8; 33.6)**	**37 (33.1; 40.9)**
WAZ	-1.40 (-1.54; -1.25)	-1.47 (-1.61; -1.34)	-1.69 (-1.84; -1.53)	-1.61 (-1.69; 1.53)	**-1.46* (-1.59; -1.33)**	**-1.66 (-1.80; -1.52)**

‡ mean with 95% confidence intervals in parentheses (controlling for population and design effect)
* p-value<0.05; ** p-value<0.01; *** p-value<0.001 (within time across group comparison)

Table S5. Crude regression on nutrition outcome indicators by sample (2012, 2014, and 2015).

Sample	Outcome indicator	Time	treatment	Time* treatment	# of children<5	constant	n
Non-matched	Wasting	0.07 (-0.04; 0.18)	0.05 (-0.30; 0.41)	-0.09 (-0.27; 0.08)		-1.76*** (2.00; -1.51)	3462
	Severe wasting	**0.35*** **(0.03; 0.67)**	0.58 (-0.21; 1.37)	-0.45 (-0.93; 0.02)		-3.82*** (-4.47; -3.18)	3462
	WHZ	**-0.04*** **(-0.08; -0.00)**	-0.00 (-0.16; 0.14)	**0.06*** **(0.00; 0.13)**		-0.88*** (-0.99; -0.78)	3462
	Stunting	0.04 (-0.01; 0.11)	-0.04 (-0.39; 0.31)	-0.00 (-0.15; 0.13)		-0.42*** (-0.58; -0.25)	3459
	HAZ	-0.05 (-0.10; 0.00)	0.03 (-0.23; 0.29)	0.03 (-0.14; 0.07)		-1.50*** (-1.66; -1.34)	3459
	Under-weight	**0.08**** **(0.02; 0.14)**	-0.03 (-0.34; 0.27)	-0.07 (-0.20; 0.04)		-0.76*** (-0.97; -0.55)	3522
	WAZ	**-0.06***** **(-0.09; -0.31)**	0.03 (-0.15; 0.22)	0.01 (-0.05; 0.09)		-1.48*** (-1.59; -1.35)	3522
Matched on the hh (worst-off child)	Wasting	0.02 (-0.09; 0.15)	0.18 (-0.24; 0.62)	-0.14 (-0.32; 0.03)	**0.48***** **(-0.32; 0.65)**	-2.53*** (-2.99; -2.07)	2254
	Severe wasting	**0.28*** **(0.05; 0.51)**	**0.86*** **(0.20; 1.53)**	**-0.63***** **(-0.96; -0.30)**	**0.55***** **(0.29; 0.81)**	-4.80*** (-5.72; -3.89)	2254
	WHZ	-0.01 (-0.06; 0.02)	-0.06 (-0.25; 0.11)	**0.08**** **(0.02; 0.14)**	**-0.25***** **(-0.31; -0.19)**	-0.72*** (-0.88; 0.55)	2258
	Stunting	-0.03 (-0.06; 0.13)	-0.24 (-0.56; 0.07)	0.06 (-0.07; 0.20)	**0.34***** **(0.21;0.47)**	-0.62*** (-0.94; -0.30)	2261
	HAZ	0.03 (-0.09; 0.03)	0.08 (-0.14; 0.31)	-0.04 (-0.14; 0.05)	**-0.26***** **(-0.35; -0.17)**	-1.45 (-1.67; -1.22)	2261
	Under-weight	0.09 (-0.00; 0.19)	0.00 (-0.34; 0.35)	-0.08 (-0.23; 0.06)	**0.36***** **(0.22; 0.50)**	-1.14*** (-1.50; -0.78)	2277
	WAZ	-0.03 (-0.08; 0.00)	0.01 (-0.15; 0.19)	0.01 (-0.04; 0.08)	**-0.20***** **(-0.26; -0.13)**	-1.38*** (-1.55; -1.21)	2277
Matched on the child	Wasting	0.05 (-0.13; 0.25)	0.38 (-0.28; 1.05)	-0.24 (-0.52; 0.03)		-2.14*** (-2.66; -1.62)	1487
	Severe wasting	0.36 (-0.02; 0.75)	**1.26*** **(0.11; 2.41)**	**-0.88***** **(-1.41; -0.35)**		-4.05*** (-5.27; -2.83)	1487
	WHZ	-0.03 (-0.10; 0.02)	-0.13 (-0.39; 0.12)	**0.10*** **(0.01; 0.20)**		-0.89*** (-1.07; 0.70)	1487
	Stunting	0.05 (-0.09; 0.21)	-0.05 (-0.61; 0.46)	0.05 (-0.15; 0.27)		-0.59** (-0.97; 0.21)	1487
	HAZ	-0.07 (-0.16; 0.09)	-0.12 (-0.44; 0.19)	0.02 (-0.10; 0.14)		-1.46*** (-1.68; -1.23)	1487
	Under-weight	**0.20*** **(0.03; 0.37)**	0.52 (-0.07; 1.12)	**-0.30*** **(-0.54; -0.06)**		-1.23*** (-1.55; -0.40)	1505
	WAZ	-0.07* (-0.13; 0.00)	-0.13 (-0.36; 0.09)	0.06 (-0.02; 0.15)		-1.42*** (-1.58; -1.26)	1505

‡ coefficient with confidence intervals in parentheses (controlling for population and design effect); **bold** numbering means it is significant at p-value<0.05

* p-value<0.05; ** p-value<0.01; *** p-value<0.001 (within time across group comparison)

Table S6. Descriptive statistics for nutrition outcome indicators for matched (child and household) data.

category	indicator	*2012* Treatment	*2012* control	*2014* treatment	*2014* control	*2015* treatment	*2015* control
wasting (%)	Worst off child	21.0 (15.5; 2646)	21.3 (16.8; 25.8)	22.5 (18.0; 26.9)	20.3 (13.9; 26.6)	**18.0*** **(12.0; 24.1)**	**27.1** **(21.3; 33.0)**
	Average child	14.2 (10.2; 18.3)	15.3 (12.3; 18.3)	15.4 (11.7; 19.0)	13.7 (9.2; 18.2)	12.8 (8.3; 17.3)	17.9 (14.7; 21.1)
	Best off child	7.9 (4.3; 11.4)	9.6 (7.1; 12.1)	9.4 (4.9; 13.9)	7.6 (4.0; 11.2)	7.5 (3.4; 11.6)	10.7 (8.3; 13.0)
	Matched child	15.5 (10.0; 21.0)	12.7 (6.7; 18.6)	11.8 (7.1; 16.6)	13.6 (7.6; 16.6)	13.4 (8.9; 17.9)	17.3 (11.5; 23.1)
Severe wasting (%)	Worst off child	5.5 (2.9; 8.1)	3.1 (1.4; 4.8)	5.2 (2.0; 8.3)	6.5 (3.1; 9.8)	**3.0*** **(0.4; 5.5)**	**9.6** **(4.1; 15.1)**
	Average child	3.2 (1.6; 4.8)	3.2 (1.6; 4.8)	2.2 (0.9; 3.6)	4.4 (2.0; 6.8)	**1.9*** **(0.2; 3.6)**	**5.6** **(2.5; 8.7)**
	Best off child	1.1 (0.2; 2.1)	1.5 (0.0; 2.9)	0	2.5 (0.4; 4.6)	0.6 (0.3; 1.6)	3.0 (0.6; 5.3)
	Matched child	4.9 (1.9; 8.0)	2.0 (0.4; 4.5)	1.3 (0.4; 3.0)	4.9 (1.8; 8.0)	2.4 (0.0; 5.0)	4.8 (2.3; 7.2)
WHZ	Worst off child	-1.20 (-1.36; -1.05)	-1.17 (-1.27; -1.07)	-1.15 (-1.29; -1.01)	-1.23 (-1.39; -1.06)	**-1.04**** **(-1.20; -0.88)**	**-1.40** **(-1.61; -1.19)**
	Average child	-0.88 (-1.01; -0.75)	-0.93 (-1.03; -0.83)	-0.83 (-0.93; -0.73)	-0.92 (-1.06; -0.77)	**-0.77*** **(-0.94; -0.60)**	**-1.07** **(-1.23; -0.91)**
	Best off child	-0.55 (-0.70; -0.41)	-0.69 (-0.80; -0.58)	-0.51 (-0.64; -0.39)	-0.60 (-0.75; -0.46)	**-0.50*** **(-0.70; -0.30)**	**-0.78** **(-0.92; -0.64)**
	Matched child	-0.96 (-1.18; -0.74)	-0.83 (-1.03; -0.63)	-0.79 (-0.96; -0.63)	-0.91 (-1.08; -0.75)	**-0.81*** **(-1.00; -0.63)**	**-1.07** **(-1.21; -0.93)**
stunting (%)	Worst off child	47.3 (39.6; 54.9)	49.2 (44.4; 54.0)	57.2 (49.3; 65.1)	55.1 (50.4; 59.8)	47.2 (41.0; 53.4)	52.0 (47.2; 56.8)
	Average child	36.5 (29.7; 43.3)	41.4 (35.7; 47.1)	45.0 (38.7; 51.3)	44.4 (39.8; 49.0)	37.7 (31.3; 44.1)	42.0 (36.8; 47.2)
	Best off child	26.5 (20.1; 33.0)	34.4 (27.3; 41.6)	32.9 (27.3; 38.5)	34.3 (29.8; 38.8)	28.5 (20.9; 36.0)	32.4 (26.7; 38.0)
	Matched child	40.8 (30.4; 51.3)	37.5 (30.7; 44.3)	46.8 (39.4; 54.2)	43.2 (36.4; 50.1)	40.2 (32.8; 47.6)	42.7 (35.3; 50.1)
HAZ	Worst off child	-1.83 (-2.04; -1.63)	-1.93 (-2.12; -1.73)	-2.30 (-2.55; -2.04)	-2.14 (-2.31; -1.96)	-2.00 (-2.14; -1.85)	-1.97 (-2.11; -1.84)
	Average child	-1.40 (-1.59; -1.21)	-1.56 (-1.77; -1.35)	-1.86 (-2.09; -1.64)	-1.73 (-1.89; -1.56)	-1.62 (-1.76; -1.48)	-1.58 (-1.72; -1.44)
	Best off child	-0.97 (-1.18; -0.77)	-1.20 (-1.44; -0.96)	-1.42 (-1.65; -1.20)	-1.31 (-1.51; -1.12)	-1.25 (-1.42; -1.08)	-1.21 (-1.37; -1.05)
	Matched child	-1.61 (-1.95; -1.27)	-1.41 (-1.61; -1.20)	-1.96 (-2.20; -1.71)	-1.71 (-1.88; -1.53)	-1.62 (-1.84; -1.40)	-1.69 (-1.92; -1.46)
underweight (%)	Worst off child	39.7 (33.4; 45.9)	39.8 (34.5; 45.0)	45.3 (36.8; 53.8)	48.8 (43.5; 54.1)	**36.6*** **(30.2; 43.0)**	**47.1** **(41.5; 52.7)**
	Average child	30.3 (25.0; 35.6)	33.2 (28.1; 38.4)	34.4 (27.3; 41.6)	39.0 (34.4; 43.7)	**28.3*** **(23.0; 33.6)**	**36.4** **(32.1; 40.7)**
	Best off child	21.4 (16.0; 26.7)	26.8 (21.6; 32.0)	23.8 (16.6; 31.0)	29.5 (24.6; 34.4)	**20.3*** **(15.4; 25.3)**	**27.8** **(22.5; 31.5)**
	Matched child	**39.6*** **(30.8; 48.4)**	**25.9** **(17.3; 34.4)**	32.7 (23.1; 42.3)	38.1 (33.3; 42.9)	**29.8*** **(23.2; 36.3)**	**40.8** **(34.2; 47.5)**
WAZ	Worst off child	-1.70	-1.75	-1.95	-1.95	**-1.71***	**-1.94**

	(-1.86; -1.54)	(-1.88; -1.63)	(-2.15; -1.76)	(-2.05; -1.85)	**(-1.85; -1.56)**	**(-2.1; -1.79)**
Average child	-1.40	-1.52	-1.64	-1.65	**-1.45***	**-1.63**
	(-1.55; -1.25)	(-1.66; -1.37)	(-1.80; -1.48)	(-1.75; -1.56)	**(-1.58; -1.32)**	**(-1.77; -1.50)**
Best off child	-1.10	-1.29	-1.33	-1.36	-1.20	-1.35
	(-1.26; -0.93)	(-1.46; -1.12)	(-1.49; -1.17)	(-1.48; -1.25)	(-1.34; -1.05)	(-1.50; -1.20)
Matched child	-1.60	-1.35	-1.65	-1.62	-1.51	-1.68
	(-1.80; -1.39)	(-1.55; -1.16)	(-1.82; -1.48)	(-1.73; -1.51)	(-1.66; -1.37)	(-1.86; -150)

‡ mean with 95% confidence intervals in parentheses (controlling for population and design effect)

* p-value<0.05; ** p-value<0.01; *** p-value<0.001 (within time across group comparison)

Table S7. Regression on nutrition outcome indicators for matched household data: worst-off and best-off-child (2012, 2014, 2015) only for households that have 2 or more children.

Sample	Outcome indicator	time	treatment	Time* treatment	# of children<5	Female child	Age (in months)	Child<5 died in hh	constant	N
Worst off child	Wasting	0.09<br>(-0.07; 0.27)	0.28<br>(-0.29; 0.86)	-0.19<br>(-0.44; 0.05)	0.26<br>(-0.02; 0.56)	-0.03<br>(-0.29; 0.36)	0.01*<br>(0.00; 0.02)	**-1.12***<br>**(-2.14; -0.09)**	-2.32***<br>(-3.25; -1.38)	1100
	Severe wasting	**0.32***<br>**(-0.01; 0.63)**	**1.12***<br>**(0.23; 2.02)**	**-0.61****<br>**(-1.04; -0.19)**	**0.52***<br>**(0.12; 0.91)**	-0.09<br>(-0.40; 0.59)	-0.01<br>(-0.00; 0.02)	0.00<br>(-1.32; 1.32)	-5.25***<br>(-6.90; -3.60)	1100
	WHZ	0.04<br>(-0.10; 0.02)	-0.15<br>(-0.38; 0.07)	0.07<br>(-0.01; 0.16)	**-0.16****<br>**(-0.27; -0.05)**	-0.04<br>(-0.16; 0.07)	-0.00**<br>(-0.01; -0.00)	0.00<br>(-0.30; 0.30)	-0.81***<br>(-1.15; -0.48)	1100
	Stunting	-0.08<br>(-0.06; 0.23)	0.00<br>(-0.45; 0.46)	0.07<br>(-0.13; 0.29)	-0.04<br>(-0.29; 0.21)	-0.27<br>(-0.55; 0.00)	-0.00<br>(-0.01; 0.00)	0.67<br>(-0.10; 1.44)	0.56<br>(-0.20; 1.33)	1100
	HAZ	0.04<br>(-0.13; 0.04)	-0.08<br>(-0.36; 0.19)	-0.02<br>(-0.15; 0.11)	-0.00<br>(-0.16; 0.15)	**0.20***<br>**(0.03; 0.37)**	0.00<br>(-0.00; 0.01)	-0.35<br>(-0.80; 0.08)	-2.41<br>(-2.88; -1.94)	1100
	Under-weight	**0.22****<br>**(0.06; 0.38)**	0.35<br>(-0.12; 0.83)	-0.11<br>(-0.33; 0.11)	0.13<br>(-0.14; 0.40)	**-0.39****<br>**(-0.69; -0.09)**	**-0.01****<br>**(-0.02; -0.00)**	0.60<br>(-0.17; 1.39)	-0.08<br>(-0.88; 0.70)	1100
	WAZ	**-0.07***<br>**(-0.13; -0.00)**	-0.12<br>(-0.33; 0.09)	0.02<br>(-0.06; 0.12)	-0.09<br>(-0.20; 0.02)	**0.19****<br>**(0.06; 0.32)**	**0.00***<br>**(0.00; 0.00)**	-0.22<br>(-0.54; 0.10)	-1.97***<br>(-2.31; -1.63)	1100
Best off child	Wasting	0.30<br>(-0.11; 0.72)	0.47<br>(-0.85; 1.81)	-0.26<br>(-0.83; 0.31)	-0.27<br>(-0.98; 0.44)	-0.38<br>(-1.13; 0.35)	0.00<br>(-0.01; 0.03)	-0.78<br>(-3.16; 1.59)	-4.31<br>(-6.70; -1.91)	1100
	Severe wasting	1.47<br>(-0.42; 3.37)	2.73<br>(-3.05; 8.52)	-1.54<br>(-3.73; 0.64)	-0.06<br>(-1.50; 1.37)	0.89<br>(-0.76; 2.55)	0.04<br>(-0.01; 0.10)	-13.0<br>(-1582; 1556)	-10.3<br>(-17.2; -3.49)	1100
	WHZ	**-0.04**<br>**(-0.11; -0.01)**	-0.00<br>-0.25; 0.26)	-0.01<br>(-0.10; 0.08)	0.05<br>(-0.05; 0.17)	0.07<br>(-0.19; 0.05)	**-0.00****<br>**(-0.01; -0.00)**	0.05<br>(-0.27; 0.37)	-0.16<br>(-0.53; 0.19)	1100
	Stunting	0.00<br>(-0.18; 0.18)	-0.25<br>(-0.87; 0.35)	0.21<br>(-0.05; 0.48)	**-0.42***<br>**(-0.78; -0.07)**	0.08<br>(-0.42; 0.25)	-0.00<br>(-0.01; 0.00)	0.36<br>(-0.44; 1.17)	-0.61<br>(-1.56; 0.33)	1110
	HAZ	**-0.06**<br>**(-0.15; 0.02)**	-0.00<br>(-0.29; 0.28)	-0.09<br>(-0.22; 0.03)	**0.30*****<br>**(0.14; 0.46)**	-0.01<br>(-0.18; 0.16)	**-0.00****<br>**(-0.01; -0.00)**	0.08<br>(-0.36; 0.53)	-0.94***<br>(-1.40; -0.47)	1110
	Under-weight	**0.14**<br>**(-0.08; 0.37)**	-0.04<br>(-0.80; 0.70)	0.00<br>(-0.32; 0.33)	-0.31<br>(-0.72; 0.09)	-0.63**<br>(-1.07; -0.18)	**-0.00**<br>**(-0.01; 0.01)**	0.49<br>(-0.50; 1.50)	-1.57<br>(-2.75; -0.39)	1100
	WAZ	**-0.05**<br>**(-0.11; 0.00)**	0.04<br>(-0.16; 0.24)	-0.05<br>(-0.13; 0.02)	**0.16****<br>**(0.10; 0.30)**	0.14*<br>(0.03; 0.26)	**-0.00**<br>**(-0.00; 0.00)**	-0.00<br>(-0.29; 0.29)	-1.34<br>(-1.65; -1.04)	1100

‡ coefficient with confidence intervals in parentheses (controlling for population and design effect); **bold** numbering means it is significant at p-value<0.05
* p-value<0.05; ** p-value<0.01; *** p-value<0.001 (within time across group comparison)
